# Whole-genome probe capture sequencing reveals genomic diversity and characteristics of *Mycoplasma pneumoniae* in Nanjing, China

**DOI:** 10.3389/fmicb.2025.1589971

**Published:** 2025-05-14

**Authors:** Guilan Zhou, Yan Yu, Teng Ge, Chenyu Tang, Hongbo Zhang, Min He

**Affiliations:** ^1^Nanjing Center for Disease Control and Prevention, Nanjing, Jiangsu, China; ^2^Nanjing Municipal Key Laboratory for Public Health Laboratory Technology, Nanjing, Jiangsu, China

**Keywords:** *Mycoplasma pneumoniae*, probe capture sequencing, genomic characteristics, P1 genotype, 23S rRNA gene

## Abstract

*Mycoplasma pneumoniae* (*M. pneumoniae*), a slow-growing, fastidious Gram-negative bacterium and a leading cause of community-acquired pneumonia globally, remains understudied and underreported across numerous geographical areas in China despite its worldwide significance. This study employed probe capture sequencing for targeted enrichment and direct sequencing of *M. pneumoniae* from clinical samples, combined with comparative genomic analyses of contemporary and historical global genomes. Core genome and pan-genome revealed that the *M. pneumoniae* genomes were classified into two distinct clades, P1-I and P1-II, each associated with a specific sequence type (ST). Most of the genomes sequenced in this study were identified as P1-I (86.96%, 20/23), contrasting with the previously reported predominance of P1-II in the area. A limited number of single-nucleotide variations were identified in the virulence-associated genes between P1-I and P1-II, leading to amino acid substitutions. The A2063G point mutation in the 23S rRNA gene was detected in all sequenced genomes (23/23), demonstrating a 100% mutation rate. This study provides the first reported application of probe capture methodology for *M. pneumoniae*, highlighting the critical importance of sustained surveillance efforts to monitor the evolution and epidemiology of this pathogen.

## Introduction

*Mycoplasma pneumoniae* (*M. pneumoniae*) is recognized as a significant causative agent of respiratory tract infections in pediatric and adult populations, encompassing a spectrum of clinical manifestations ranging from mild upper respiratory infections to severe, life-threatening conditions (Paquette et al., 2024). Infections caused by *M. pneumoniae* were generally self-limiting; however, progression to severe pneumonia was documented in some cases (Youn and Lee, 2012; Paquette et al., 2024). Macrolide antibiotics were commonly used as the first-line treatment for *M. pneumoniae* infections (Gao and Sun, 2024). Nevertheless, since 2000, the emergence of macrolide-resistant *M. pneumoniae* strains has been reported globally, with varying resistance rates and a notably higher prevalence observed in East Asia, particularly China and Japan (Tanaka et al., 2017; Kim et al., 2022; Chen et al., 2024a; Li et al., 2024). Molecular characterization of macrolide resistance in *M. pneumoniae* has identified nucleotide substitutions (predominantly A-G transitions) at positions 2063 or 2064 in domain V of the 23S rRNA gene as the principal determinants of high-level resistance (Lucier et al., 1995; Matsuoka et al., 2004; Pereyre et al., 2016; Kim et al., 2022). The C2617A/T mutation and single amino acid changes (G72R, G72V) in ribosomal protein L4 have also been reported in the literature (Wang et al., 2023).

The pathogenic mechanisms of *M. pneumoniae* remained under active investigation, with several key virulence factors having been identified, including the P1 protein within the adhesin complex and the Community-Acquired Respiratory Distress Syndrome (CARDS) toxin (Rodman Berlot et al., 2021; Wang et al., 2022). The P1 adhesin (P1), a 170-kD surface protein localized to the terminal structure of pathogenic *M. pneumoniae*, has been utilized for classifying *M. pneumoniae* into two subtypes, P1-I and P1-II, based on sequence polymorphisms (Sasaki et al., 1996). Additionally, novel genetic typing technologies, such as Multiple-Locus Variable-number tandem repeat Analysis (MLVA) and Multilocus Sequence Typing (MLST), have been successfully implemented for molecular typing of *M. pneumoniae* due to their superior resolution and reproducibility (Sun et al., 2013; Chalker et al., 2015; Lu et al., 2020). Genetic studies on *M. pneumoniae* mainly focus on the P1 genotype and subtype (Su et al., 1988; Su et al., 1990; Kenri et al., 2008). The CARDS toxin, an ADP-ribosylating and vacuolating toxin, has been demonstrated to induce pulmonary inflammation and airway hyperreactivity (Medina et al., 2012; Medina et al., 2014). Notably, a difference in the CARDS toxin-related gene has been identified between P1-I and P1-II, although its impact on the toxin’s functional properties remains unclear (Xiao et al., 2015). Studies have demonstrated that the P1-I and P1-II genotypes exhibit clonal characteristics, showing remarkable overall similarity among *M. pneumoniae* genomes with >99% sequence identity while displaying particularly striking intra-subtype similarity characterized by <0.1% genetic variation within the same subtype (Xiao et al., 2015).

Genomics provides vital information for studying the structure, composition, function, and evolution of *M. pneumoniae* on a genome-wide scale. With advancements in molecular biology and bioinformatics, the genomic analysis of *M. pneumoniae* has provided valuable insights into its epidemiology, drug resistance, and evolutionary characteristics, thereby offering evidence-based guidance for clinical practice. Most *M. pneumoniae*-related studies in mainland China were conducted in Beijing and Shanghai, while few were performed in other regions. As the capital of Jiangsu Province, Nanjing represents an ideal sentinel site for investigating *M. pneumoniae* transmission dynamics due to its unique combination of: (1) historical urban infrastructure, (2) tourism-mediated population mobility, (3) an economically developed setting, and (4) high population density. These characteristics facilitate local transmission monitoring and enable the study of potential international dissemination routes through its transportation hub, establishing Nanjing as a strategic location for respiratory pathogen surveillance in eastern China. In the present study, a comprehensive analysis of 311 *M. pneumoniae* genomes was performed, among which 23 were newly sequenced using an innovative approach combining probe-based methods and targeted capture sequencing. The technique relied on the specific hybridization of complementary nucleic acid probes to target DNA fragments, facilitating the enrichment of the desired sequences from complex genomic samples. Targeted enrichment can be helpful when the whole genome is not required or a particular genome of interest is selected from contaminating DNA (Summerer, 2009; Clark et al., 2018). Thus, the method is principal and provides an efficient alternative to cell culture combined with whole-genome sequencing for *M. pneumoniae*. This study aims to elucidate the genetic variation among *M. pneumoniae* genomes and establish a foundational reference for future research.

## Materials and methods

### Clinical information collection, next-generation sequencing, and assembly

**Figure 1:**
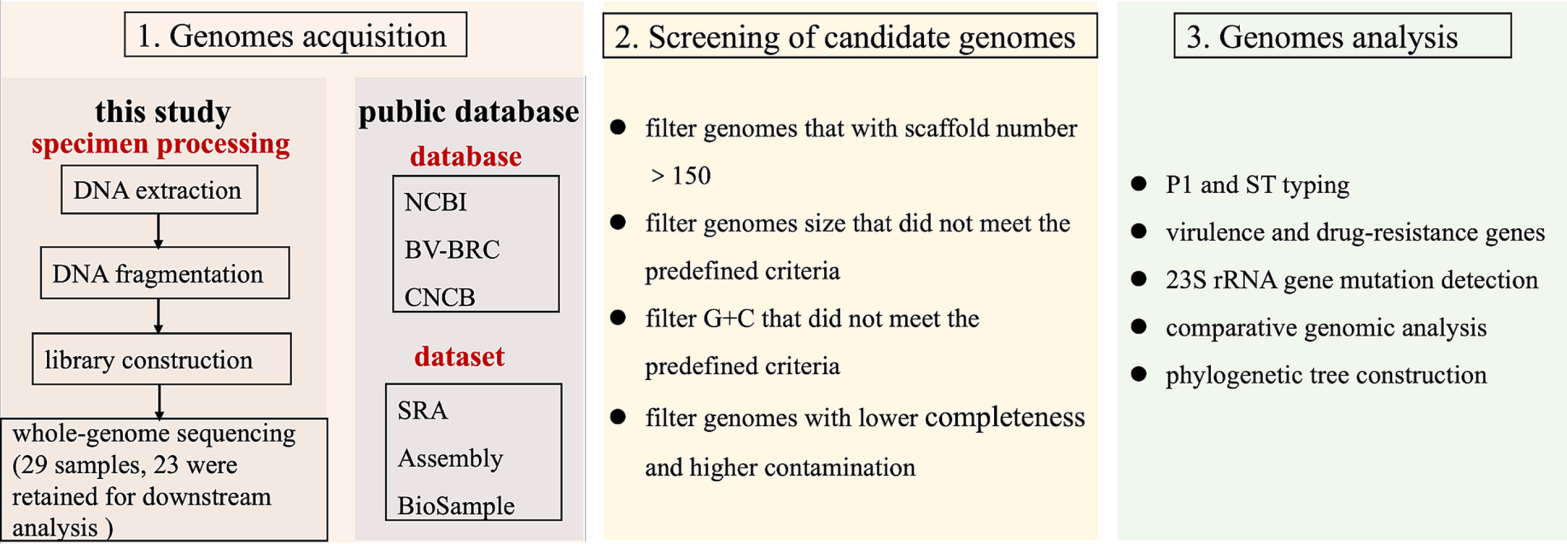
Schematic diagram of the research framework. Panel 1: genome acquisition, incorporating both newly sequenced samples and publicly available datasets. Panel 2: quality-controlled genomes, with 311 high-quality genomes retained (23 newly sequenced and 288 from public databases). Panel 3: comprehensive genomic analyses.

Twenty-nine *M. pneumoniae*-positive samples, comprising 14 bronchoalveolar lavage fluid samples, 10 throat swab samples, and 5 sputum samples, were selected from routine respiratory surveillance specimens collected in Nanjing, Jiangsu Province, China, between 2023 and 2024 for probe capture sequencing. Sample genomic DNA was first extracted from archived positive samples (−80°C) using the FastPure Bacteria DNA Isolation Mini Kit (Vazyme, China) following the manufacturer’s instructions. All obtained DNA samples met the minimum quantity threshold of >5 ng. The extracted DNA was then enzymatically fragmented and converted into sequencing libraries using the PK10003NM-12-MGI kit (Homgen Co., Ltd., China) according to the recommended protocol. Subsequently, automated library construction was performed using the *Mycoplasma pneumoniae* probe-based whole-genome capture and library preparation kit P10020-12 (homgen Co., Ltd., China) by the manufacturer’s protocol. Finally, whole-genome sequencing was conducted on the Illumina MiSeq platform (Figure 1). Paired-end sequencing was performed with a read length of 150 bp, achieving an average sequencing depth of 463 × (ranging from 140 × to 2,142×). Quality control of the sequencing data was conducted using FastQC v0.12.1 software.^1^ The reads were trimmed using fastp v0.24.0 software (Chen, 2023) and subsequently assembled using SPAdes v4.0.0 software (Prjibelski et al., 2020). The quality of the assembled genomes, including genome size, scaffolds’ count, N50, G + C content, predicted genes, contamination, and completeness, was evaluated using CheckM v1.2.3 software.^2^ Genomic annotation was performed using a locally installed prokka v1.14.6 software (Seemann, 2014) to predict protein-coding sequences and tRNAs. Ribosomal RNA genes were annotated using Barrnap v0.9 software,^3^ which was integrated into the prokka pipeline. In this study, 29 genomes were successfully sequenced, among which six were excluded from further analysis for the following reasons: insufficient sequencing depth was detected in some samples upon analysis of the raw sequencing data. Furthermore, poor sequence completeness was observed after *de novo* genome assembly. Importantly, these genomes were not confirmed as the target species (*M. pneumoniae*) based on the species identification using GTDB-Tk v2.3.2 software (Chaumeil et al., 2022).

### Public data download

All genome sequences identified as *M. pneumoniae* were retrieved from publicly accessible databases, including the NCBI,^4^ BV-BRC,^5^ and CNCB,^6^ as of January 2025. The publicly available assemblies were subjected to quality control using Quast v5.0.2 software (Gurevich et al., 2013). Initially, species identification was performed on the obtained genomes using GTDB-Tk v2.3.2 software, and only those confirmed as *M. pneumoniae* were retained for further analysis. Subsequently, genomes that did not meet the predefined criteria for genome size and G + C content, based on the genomic characteristics of *M. pneumoniae*, were excluded. Finally, to ensure the reliability of the analysis, only genomes with fewer than 150 scaffolds were included (Figure 1). In total, 311 *M. pneumoniae* genomes were analyzed, comprising 23 sequenced in this study and 288 obtained from the databases, as summarized in Supplementary Table S1.

### P1 and ST typing

The specific short sequences of P1-I and P1-II genotypes were referenced from different studies. Through short-sequence alignment analysis, the primer and probe sequences provided by Wolff et al. (2017) (P1-I_F: 5’-GGTATAATTGTTTGGATTCGCC-3′; P1-I_R: 5’-CTTACGATTCCAGTAGCATTAAGATTC-3′; P1-I_probe: 5’-TAAATGGAAGACAATTAAATGGAAGACAAT-3′; P1-II_F: 5’-GGGGAACCTTACTTTTGTTTGT-3′; P1-II_R: 5’-GCCTTAATTGTTGGCATCATTAAT-3′; P1-II_probe: 5’-CATGCTAATTAAGTAGTAGCACAGAA-3′) were tested to be entirely consistent with the experimentally verified P1 genotyping results reported in the corresponding literature. Therefore, these sequences were adopted as the standard reference for P1 genotyping in the present study. The genome sequence (accession number GCF_002147855) and annotation information of the P1-I reference genome M129 and the raw sequencing data (accession number SRR3924617) were obtained from the NCBI database. Similarly, the genome sequence (accession number GCF_001272835) and the P1-II reference genome FH annotation information were also retrieved from the NCBI database. Sequence type (ST) confirmation was performed using mlst v2.23.0 software^7^ and the pubMLST database.^8^ The minimum spanning tree (MST) was constructed using the PHYLOViZ Online platform.^9^

### Detection of virulence genes and drug-resistance genes

Virulence genes across all genomes were identified using VFanalyzer.^10^ Resistance genes were predicted through Abricate v1.0.1 software^11^ and the Comprehensive Antibiotic Resistance Database (CARD),^12^ with an E-value threshold of 1e-5 applied as the cutoff. Individual missense mutations in the 23S rRNA gene, known to confer macrolide resistance, were detected using BLAST v2.12.0 and MAFFT v7.520 software.^13^

### Evolutionary tree construction

Whole-genome alignments for single-nucleotide polymorphism (SNP) variant sites were generated using Snippy v4.6.0 software,^14^ with the M129 genome (NCBI accession number GCF_002147855) serving as the reference genome. Recombinant sites were removed using Gubbins v2.3.4 software (Croucher et al., 2015) under default settings. A maximum likelihood (ML) tree was built using FastTree v2.1 software (Price et al., 2010), employing the GTR + T (gamma) model and 1,000 bootstrap replicates. The phylogenetic tree was visualized and annotated using the Interactive Tree of Life (iTOL)^15^ platform and the table2itol.R package.

### Comparative genomic analysis

Genome sequence similarity between genomes was determined using chromatiblock v1.0.0 software (Sullivan and van Bakel, 2020). Multiple-sequence alignment was presented by the WebLogo website.^16^ Differential genes among the genomes were identified through a comparative analysis of genome annotation files using Roary v3.12.0 software (Page et al., 2015). Specifically, each genome’s genomic feature files generated by prokka in gff3 format were utilized for pan-genome analysis. Roary was executed with the “-e” flag to produce a multi-FASTA alignment of core genes using PRANK, with a minimum blastp identity threshold set at 95%. The pan-genome composition summary, as Roary provided, was visualized using the open-source Python script “roary_plots.py.”^17^

## Results

### Genomes sequenced in this study

Twenty-nine *M. pneumoniae*-positive samples were collected through routine respiratory surveillance and processed using probe capture sequencing. Of these, 23 samples yield high-quality sequencing quality data. The assembly characteristics and genomic features of each genome included in this study were summarized in Table 1. The sequenced *M. pneumoniae* genomes demonstrated exceptional quality, with completeness ≥89.4%, contamination levels ≤1.0%, and an average of approximately 46 scaffolds per genome. The genome sizes ranged from 809,750 to 894,235 bp, and the G + C content varied between 39.8 and 40.6%. Comparative genomic analysis revealed that these genomic characteristics showed high concordance with reference genomes available in public databases (Supplementary Table S1).

**Table 1:** Overview of sequenced genomes and supplementary information used in this study.

Sample	Collect date	Sample type	Sequence depth	Genome size	G + C	Scaffold	Completeness	Contamination	Predicted genes	Mutation in 23S rRNA	*aph(3″)-Ib*	*aph(6)-Id*	*blaTEM-1*	*blaTEM-150*	*blaTEM-235*	MLST	P1 Typing
NJMP202303	2023	Throat swab	157	859836	39.85%	52	99.25%	0.00%	841	A2063G					+	3	I
NJMP202306	2023	Alveolar lavage fluid	141	886310	40.10%	50	100%	0.00%	848	A2063G					+	3	I
NJMP202310	2023	Alveolar lavage fluid	173	894235	40.12%	48	99.25%	0.00%	875	A2063G					+	3	I
NJMP202311	2023	Alveolar lavage fluid	234	830066	40.15%	119	89.41%	0.00%	878	A2063G			+			−	II
NJMP202312	2023	Alveolar lavage fluid	397	863202	40.03%	42	100%	0.00%	816	A2063G						3	I
NJMP202313	2023	Alveolar lavage fluid	722	826893	39.82%	38	99.22%	0.00%	796	A2063G						3	I
NJMP202314	2023	Alveolar lavage fluid	806	853612	39.98%	44	99.25%	0.00%	814	A2063G			+			3	I
NJMP202315	2023	Alveolar lavage fluid	589	835626	39.84%	45	100%	0.00%	802	A2063G						3	I
NJMP202316	2023	Alveolar lavage fluid	577	823644	39.78%	41	99.25%	0.00%	798	A2063G						−	I
NJMP202317	2023	Alveolar lavage fluid	408	865124	39.84%	49	100%	0.00%	829	A2063G						3	I
NJMP202318	2023	Alveolar lavage fluid	2,142	864638	40.00%	42	99.25%	0.00%	831	A2063G						3	I
NJMP202319	2023	Alveolar lavage fluid	669	839700	39.83%	46	99.25%	0.00%	816	A2063G						14	II
NJMP202321	2023	Throat swab	432	882018	40.58%	35	99.25%	0.00%	823	A2063G						3	I
PINBNJ2024301	2024	sputum	472	860291	40.07%	38	99.25%	0.53%	815	A2063G				+		3	I
PINBNJ2024302	2024	sputum	164	809750	40.01%	93	96.24%	1.00%	805	A2063G				+		3	I
PINBNJ2024304	2024	sputum	351	858531	40.08%	44	99.22%	0.00%	826	A2063G					+	3	I
PINBNJ2024308	2024	sputum	461	826685	39.94%	36	99.25%	0.00%	783	A2063G					+	3	I
PINBNJ2024311	2024	Alveolar lavage fluid	279	880896	40.06%	50	100%	0.15%	858	A2063G						3	I
PINBNJ2024313	2024	Alveolar lavage fluid	567	863502	39.85%	51	99.25%	0.15%	852	A2063G						3	I
PINBNJ2024342	2024	Throat swab	148	857373	40.03%	47	99.25%	0.00%	827	A2063G	+	+	+			3	I
PINBNJ2024343	2024	Throat swab	339	869572	40.34%	42	100%	0.00%	836	A2063G		+	+			3	I
PINBNJ2024344	2024	Throat swab	148	855865	40.37%	59	100%	0.00%	843	A2063G/C2617T	+	+			+	−	II
PINBNJ2024351	2024	Throat swab	271	851882	40.06%	51	100%	0.15%	827	A2063G		+				3	I

### P1 and ST typing

**Figure 2:**
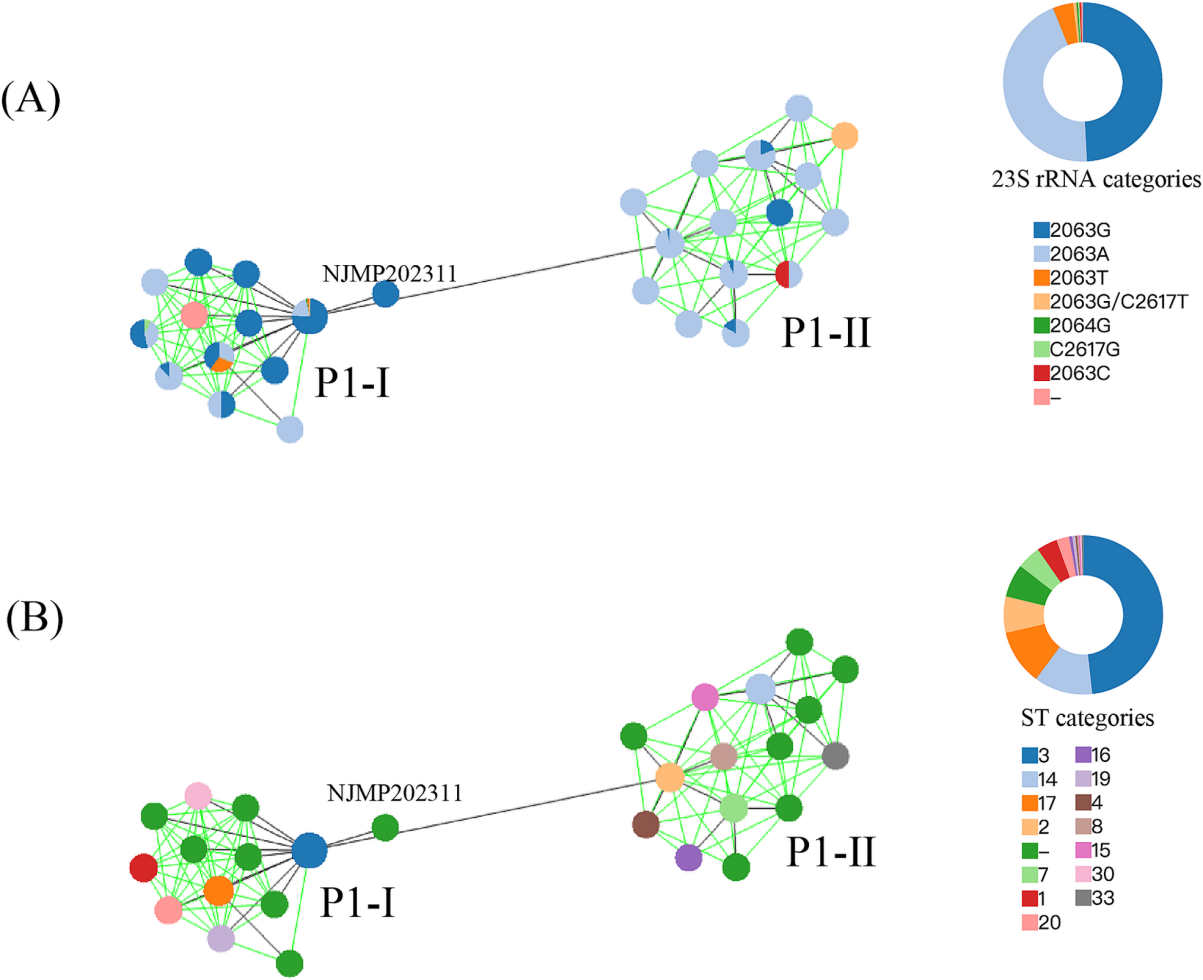
The MST was constructed based on MLST allelic profiles. A circle represented each ST, and the size of the circle was logarithmically proportional to the number of genomes with that particular ST. The right circular ring represented the proportional distribution of different classification categories. **(A)** Coloration was applied according to the mutation types at the 23S rRNA gene. **(B)** Coloration was applied according to the ST types.

Based on P1 genotyping, the 23 genomes sequenced in our laboratory were classified into 20 P1-I and 3 P1-II. The predominant sequence type identified was ST3 (19/23), with only one genome being classified as ST14, while three genomes remained untypable (Table 1). Among the 311 *M. pneumoniae* genomes analyzed, 215 were categorized as P1-I and 96 as P1-II. The MST was constructed based on MLST loci, classifying the 311 genomes into two branches, corresponding precisely to P1-I and P1-II, with NJMP202311 exhibiting unique specificity (Figure 2). The distribution of ST types was predominantly characterized by ST3 (150/311), followed by ST14 (37/311), ST17 (35/311), and ST2 (23/311). Among these, ST3 and ST17 were classified as P1-I genotypes, while ST14 and ST2 were identified as P1-II genotypes. Notably, most P1-II genomes exhibited a macrolide-sensitive characteristic (2063A), whereas P1-I genomes predominantly displayed a macrolide-resistant characteristic (2063G/C/T, 2064G, and 2617 T/G).

### Antibiotic resistance genes and virulence factors

The aminoglycoside antibiotic resistance genes, specifically *aph(3″)-Ib* and *aph(6)-Id*, were identified in four genomic sequences. In addition, the *β*-lactam antibiotic resistance genes, including *blaTEM-1*, *blaTEM-150*, and *blaTEM-235*, were detected in 5, 2, and 6 genomes, respectively. Notably, except for the GCF_009810055 genome from Japan, which harbored the *blaTEM-1* resistance gene, no other resistance genes were identified in the remaining genomes. It was noteworthy that an A-G or A-T mutation at position 2063 in the 23S rRNA gene using M129 as the reference genome, resulting in resistance to macrolide antibiotics, was detected in all the genomes sequenced in this study. In contrast, analysis of 23S rRNA genes from genomes derived from non-Chinese sources revealed a distinct mutation profile: A2063G, A2063C, A2063T, A2064G, and C2617G were observed at frequencies of 23.95% (40/167), 0.60% (1/167), 1.80% (3/167), 0.60% (1/167), and 0.60% (1/167), respectively. Comprehensive data regarding the distribution and prevalence of resistance genes across the analyzed genomes have been systematically compiled and were presented in Table 1 and Supplementary Table S1 for reference.

**Figure 3:**
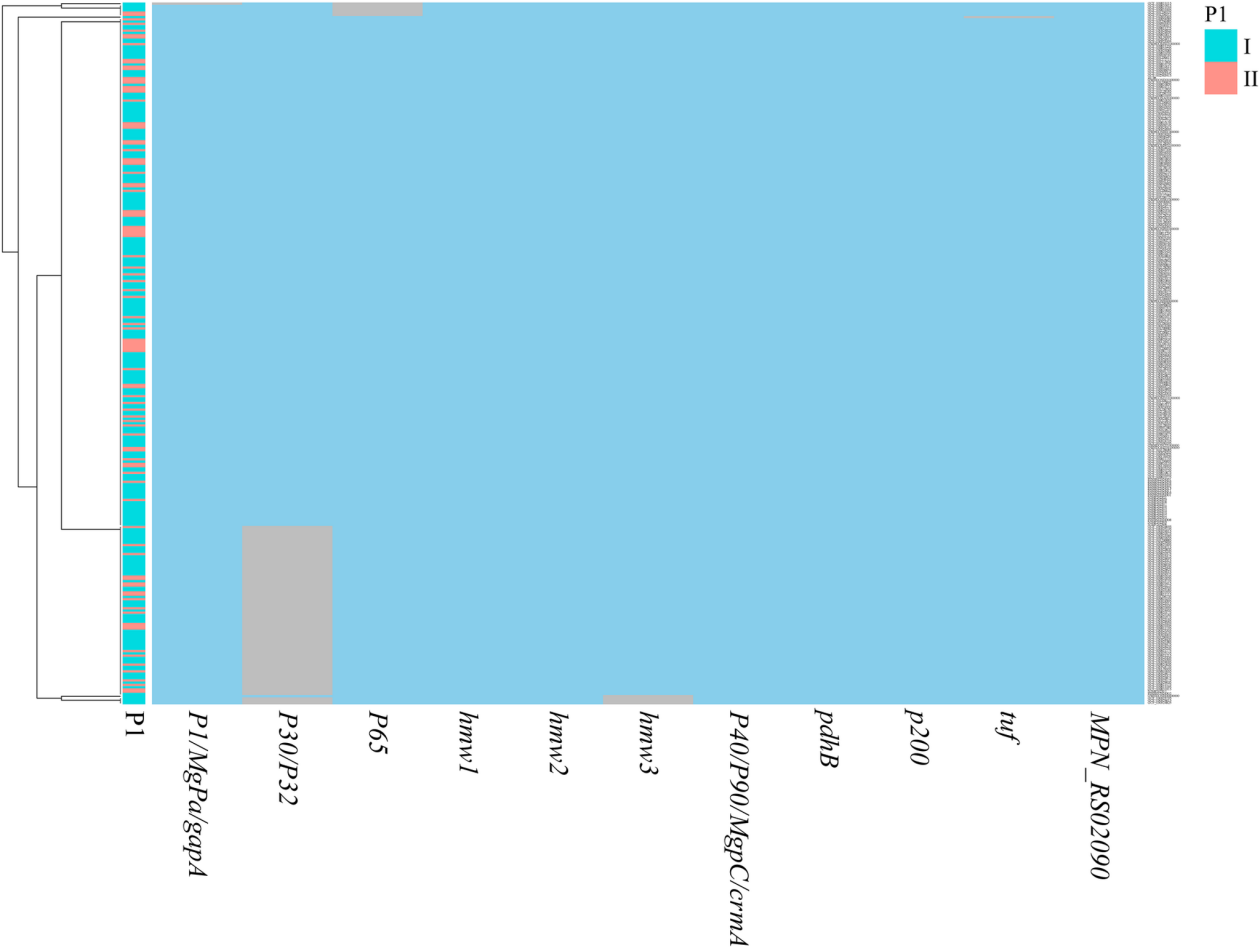
Distribution of virulence genes in *M. pneumoniae* genomes. Blue blocks represented the presence of a gene, while grey blocks indicated its absence. The left annotation bar denoted the P1 genotype for each genome.

**Figure 4:**
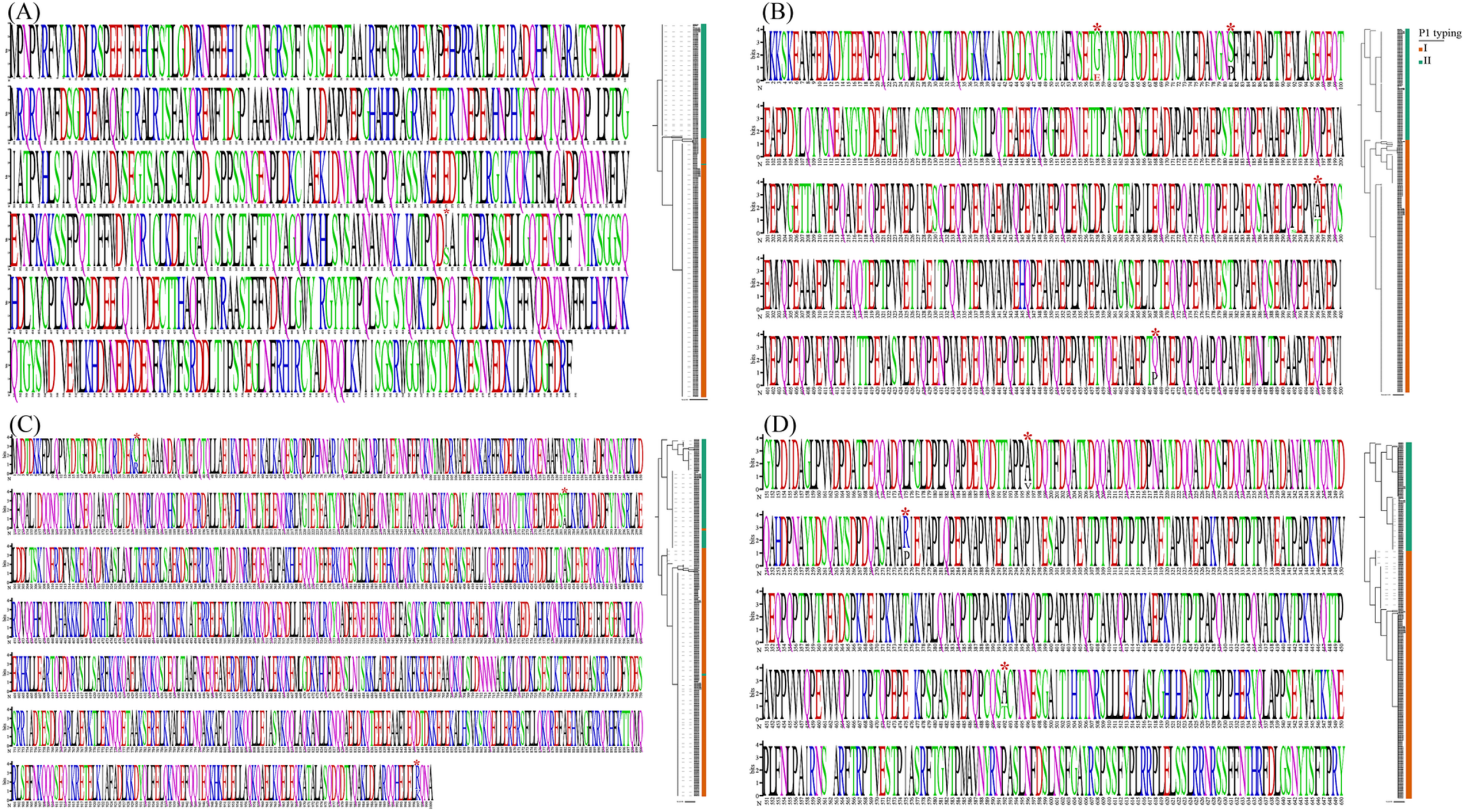
Multiple-sequence alignment and WebLogo analysis were performed on the MPN_RS02090 gene (encoding CARDS toxin), *hmw1*, *hmw2*, and *hmw3* (adhesion-related genes). The panels **(A–D)** represented the results of the MPN_RS02090, *hmw1*, *hmw2*, and *hmw3*, respectively. Left side of each panel: In the WebLogo representation, residues displayed with larger letter sizes were found to correspond to positions exhibiting a relatively high degree of conservation. The red asterisk represented amino acids showing significant differences between P1-I and P1-II. Right side of panel: An ML phylogenetic tree was constructed based on the nucleotide sequences of the MPN_RS02090, *hmw1*, *hmw2*, and *hmw3* gene.

A comprehensive array of virulence-associated genes, encompassing adhesion-related genes and the CARDS toxin, was identified across the analyzed genomes (Figure 3). Notably, the MPN_RS02090 gene encoding the CARDS toxin was found to exhibit a nucleotide variation at position 1,112 between P1-I and P1-II genomes, where the base was identified as T in P1-I and G in P1-II, resulting in amino acid substitutions of I and S, respectively. Phylogenetic analysis based on the MPN_RS02090 gene sequence revealed that the genomes could be distinctly classified into two groups, P1-I and P1-II, consistent with the classification obtained through P1 typing (Figure 4A). The results of adhesion-related genes (*hmw1*, *hmw2*, and *hmw3*) were consistent with those of the CARDS toxin gene, demonstrating high conservation in *M. pneumoniae*. Only minor amino acid variations were observed between the P1-I and P1-II genomes (Figures 4B–D).

### Genome comparison

**Figure 5:**
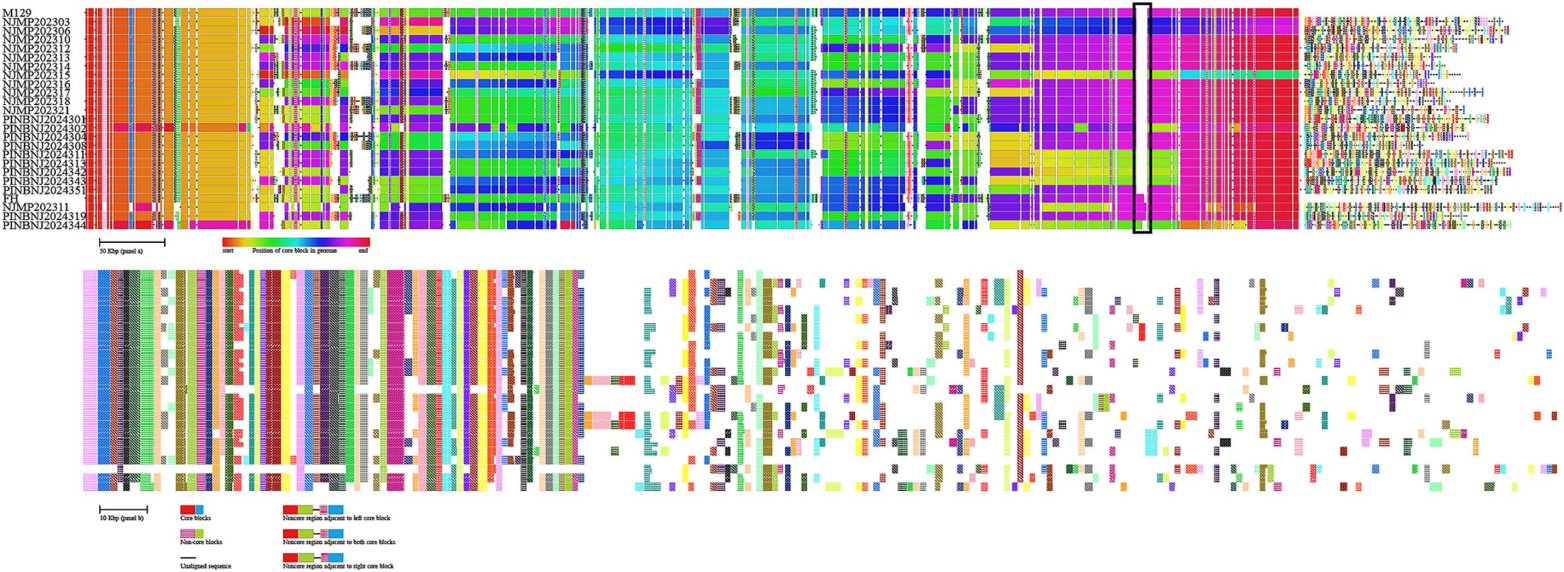
The genomic structures of 25 *M. pneumoniae* genomes were visualized using chromatiblock analysis. The sample identifiers were displayed on the left side of the visualization. (Top) Global alignment view. Core genomic blocks were represented as vertically aligned solid rectangles, with their coloration corresponding to their respective positions within the genome. Non-core blocks were depicted as patterned rectangles, where each block was assigned a unique combination of pattern and color for differentiation. Sequences exclusively unique to a single genome were illustrated as solid black lines. (Bottom) Alignment difference view. Each genome was represented as a row, and each non-core block was assigned a column in the order they were most commonly found in the genome. The presence of each non-core block was shown as a patterned rectangle in the genome row. As non-core blocks may be present more than once, duplicates were demonstrated by splitting the blocks according to repeat numbers.

The genome sequences of the 23 samples were analyzed and compared against the reference genomes M129 (P1-I) and FH (P1-II) using the chromatiblock software. Significant differences were observed in the core regions of the P1-I and P1-II genomes, particularly in the highlighted areas within the black box (Figure 5). These regions were identified as distinguishing features between the P1-I and P1-II genomes and annotated as hypothetical proteins.

### Phylogenetics of genome indices

**Figure 6:**
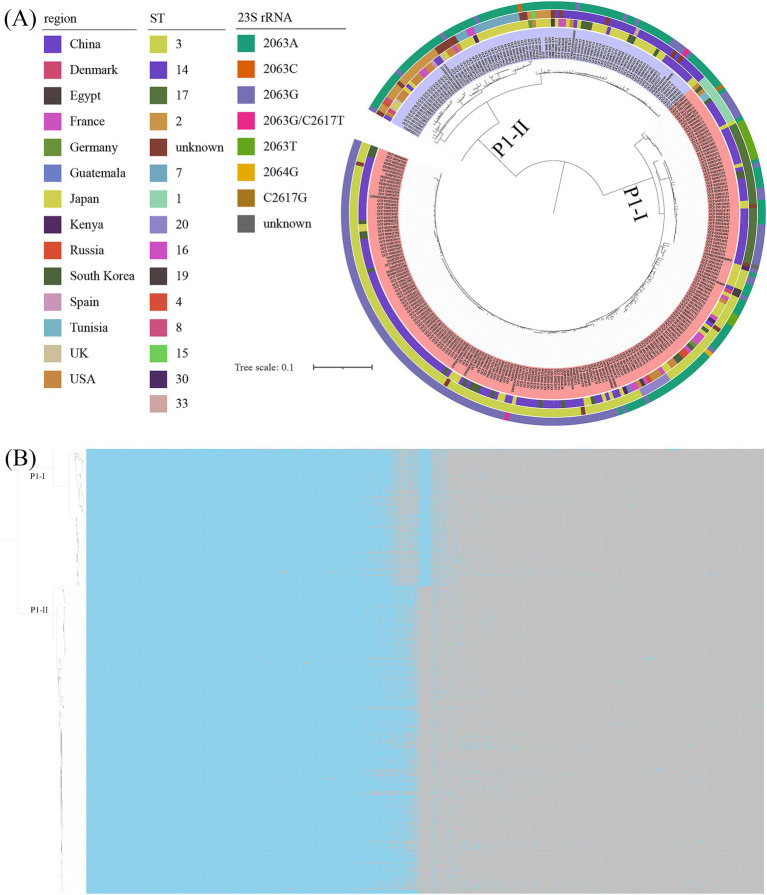
The results of the core genome and pan-genome. **(A)** The ML tree was constructed based on core genome alignments of the analyzed genomes, with branch support assessed using 1,000 bootstrap replicates. The tree was annotated with distinct colors to represent variations in geographic region, sequence type (ST), and 23S rRNA mutations. The visualization was organized into concentric rings, where the inner ring corresponded to the genomes, the first outer ring represented the geographic regions, the middle ring denoted the STs, and the outermost ring indicated the 23S rRNA mutations. Additionally, different background colors were used to distinguish between the P1 genotypes. **(B)** The results of the pan-genome analysis of 311 *M. pneumoniae* genomes were presented. Blue blocks represented the presence of a gene, while grey blocks indicated its absence. The tree on the left was an ML tree constructed based on core SNPs.

Genome-wide variation analysis was performed on all *M. pneumoniae* genomes using M129 as the reference genome, and SNP was identified for each genome. The analysis revealed that P1-II genomes exhibited significant differences from P1-I genomes. A phylogenetic tree was constructed based on the core SNP, which segregated the 311 genomes into two distinct branches corresponding to P1-I and P1-II, respectively (Figure 6A). Pairwise SNP differences among the *M. pneumoniae* genomes were visualized in the matrix shown in Fig. S1. The ST3, ST17, ST1, and ST20 were classified as P1-I genotypes, while ST14, ST2, and ST7 were identified as P1-II genotypes. Upon analyzing the distribution of resistant and susceptible genomes, the P1-II primarily displayed genotypic traits indicative of macrolide antibiotic susceptibility. Additionally, a pan-genome landscape analysis further elucidated the genomic distinctions between P1-I and P1-II genotypes (Figure 6B). The gene content (presence/absence patterns) of the 311 *M. pneumoniae* genomes was detailed in Supplementary Table S2.

## Discussion

*M. pneumoniae* is recognized as a slow-growing, fastidious, and cell wall-deficient microorganism, which is predominantly transmitted among humans through respiratory droplets produced by coughing (Atkinson et al., 2008; Paquette et al., 2024). Owing to its challenging cultivability, the isolation of pure cultures using conventional methods is often hindered, thereby restricting subsequent genomic investigations. To address this limitation, a probe capture sequencing approach was employed in this study, allowing for targeted enrichment and direct sequencing of *M. pneumoniae* genomes from clinical specimens, thereby enabling efficient and accurate genomic analysis. In this study, we conducted a comprehensive analysis of 311 *M. pneumoniae* genomes, with 23 of these sequenced by our laboratory using the probe capture sequencing method. These high-quality draft genomes were found to enrich the existing genomic database, with the majority exhibiting high levels of contiguity, completeness, and minimal contamination, thereby ensuring the reliability of the analytical outcomes.

The P1-RFLP genotyping method has been widely employed since its initial development (Sasaki et al., 1996; Xue et al., 2014; Edelstein et al., 2016; Li et al., 2022). In the present study, the genomic sequencing results revealed that the P1-I genotype was predominantly identified, accounting for 86.96% (20/23) of the total genomes sequenced by our laboratory, which was consistent with findings reported in international studies and multiple domestic investigations (Dumke et al., 2015; Xue et al., 2018; Kurkela et al., 2019; Zhao et al., 2019; Ishiguro et al., 2021; Xu et al., 2024; Li et al., 2025). However, this genotype was found to differ from the predominant strains circulating in Japan (Kenri et al., 2020; Kenri et al., 2023). Notably, the present findings were inconsistent with the results reported by Xue et al. (2018), which indicated that the P1-II genotype was the dominant subtype of *M. pneumoniae* in Nanjing. This discrepancy was hypothesized to be attributable to two major factors. Firstly, a potential bias may have been introduced due to the relatively limited sample size (n = 10) in the study by Xue et al. (2018) Secondly, significant temporal variations in predominant P1 genotypes of *M. pneumoniae* have been well documented, with this phenomenon being reported explicitly in a Japanese study; furthermore, Kenri et al. have suggested that the immune-driven genotypic shifts between P1-I and P2-II may follow a cyclical pattern (Kenri et al., 2020). Moreover, the P1 genotyping results from the Weihai region of Shandong Province revealed that the P1-II genotype was slightly predominant, accounting for 51.8% (Guo et al., 2022). This genotype transition was hypothesized to be primarily driven by antibiotic selective pressure. Macrolides were widely prescribed as first-line therapeutic agents for *M. pneumoniae* infections (Gao and Sun, 2024; Wang et al., 2024). However, a continuous increase in macrolide resistance rates among *M. pneumoniae* isolates has been observed in China in recent years (Chen et al., 2024a; Gao and Sun, 2024; Yan et al., 2024). The A2063G mutation has been demonstrated to occur at significantly higher frequencies in P1-I strains compared to P1-II strains. Consequently, under sustained antibiotic selection pressure, P1-I strains were considered to have gradually acquired a competitive growth advantage. Nevertheless, the hypothesis remained to be further validated, as the current study was limited by insufficient sample size and lacked longitudinal surveillance data. These findings further underscore the existence of significant regional variations in the distribution of the P1 genotype across different areas within the country. Consequently, to accurately delineate the epidemiological characteristics of the *M. pneumoniae* P1 genotype in other regions, the implementation of long-term, systematic molecular epidemiological surveillance was strongly recommended. Expanding the sample size and monitoring scope was essential for acquiring more representative research data.

The CARDS toxin gene and adhesion-related genes (*hmw1*, *hmw2*, and *hmw3*) were demonstrated to exhibit significantly higher conservation across all analyzed genomes than the P1 genotype, which was consistent with previous findings, suggesting its stable inheritance during evolutionary processes (Jiang et al., 2024). Only one SNP at position 1,112 of the CARDS gene was identified between the P1-I and P1-II genotypes, which was demonstrated to cause an amino acid substitution in the encoded protein. However, the functional consequences of this amino acid alteration on the toxin’s activity have not yet been fully elucidated, underscoring the need for further functional investigations of the CARDS gene. Only 2–3 amino acid differences were identified in the *hmw1*, *hmw2*, and *hmw3* genes between the P1-I and P1-II genotypes, and these differences were confirmed to exhibit significant genotype-specificity. However, whether these variations were functionally relevant has not been reported in the literature, and further investigation was required to elucidate their potential biological significance.

The genotypic resistance profiles of *M. pneumoniae* exhibited a broader spectrum of antibiotic resistance and a more significant number of resistance genes than genomes from other countries. It was noteworthy that the genomes containing antibiotic-resistance genes were almost exclusively sequenced by our laboratory. Our results revealed potentially significant differences in the antibiotic resistance patterns between *M. pneumoniae* isolates collected in Nanjing and those documented in other geographical locations. Analysis of the 23S rRNA gene in all sequenced genomes demonstrated that an A-G mutation at position 2063 was detected in 100% (23/23) of the genomes, and previous studies have indicated that mutations at this site can confer high-level resistance to macrolide antibiotics, particularly erythromycin (Kim et al., 2022). These findings suggested that *M. pneumoniae* strains in this region may exhibit a higher ratio of erythromycin resistance, representing a striking contrast to the low erythromycin resistance previously reported by Xue et al. (2018) for the same geographic area. This divergence in resistance patterns may be explained by the predominance of the P1-II genotype during the earlier study period, as the current investigation revealed that the P1-II genotype demonstrates a markedly lower mutation frequency at positions 2063 and 2064 within the 23S rRNA gene compared to the P1-I genotype. Additionally, the mutation rate at this position has been reported to be significantly lower in other countries or regions, consistent with the documented lower resistance rates of *M. pneumoniae* to macrolide antibiotics in those areas (Gullsby et al., 2019; Loconsole et al., 2019; Noori Goodarzi et al., 2020). Geographical variation in the A2063G mutation rate of *M. pneumoniae* 23S rRNA gene across Chinese regions was further supported by these findings, with the mutation frequency being observed to be significantly higher than that reported in other countries or regions (Zhao et al., 2012; Zhao et al., 2019; Lu et al., 2020; Guo et al., 2022; Chen et al., 2024b; Dekyi Xiao et al., 2024; Wang et al., 2024; Yan et al., 2024). The differences in geographic distribution may reflect ongoing species divergence or suggest transmission dynamics patterns. Additional studies were required to elucidate the implications of sequence variations in these isolates and the selective pressures driving their emergence and potential dissemination.

Phylogenetic and cluster analysis based on the MLST, core SNPs, and pan-genome revealed that the genomes could be distinctly classified into two clades, corresponding to the P1-I and P1-II typing results. Interestingly, only 6.05% (13/215) of the genomes were found to be closely related to the prototypical *M. pneumoniae* P1-I reference genome M129, suggesting that this genome was not representative of the majority of P1-I *M. pneumoniae* clinical isolates circulating worldwide. This finding aligned with conclusions previously reported by Spuesens et al. (2016) and Diaz et al. (2017), raising questions regarding the suitability of M129 as the P1-I reference genome. In contrast, FH was identified as closely related to a large subgroup of P1-II genomes (48.96%, 47/96). The MLST results revealed that among the *M. pneumoniae* genomes with known ST types, significant differences were observed between P1-I and P1-II not only in their dominant ST types but also in the complete lack of overlap in their ST type distributions, which suggested that P1-I and P1-II had undergone long-term divergence during their evolutionary processes. Furthermore, the core SNP-based phylogenetic analysis demonstrated that the mutation rate at the corresponding position of the 23S rRNA gene in P1-I genotype was significantly higher than that in P1-II, which implied that isolates of different P1 genotypes might have exhibited varying capacities to withstand antibiotic selection pressures during evolution. Intriguingly, the NJMP202311 genome was classified as a member of the P2-II genotype based on core SNP analysis, whereas it was assigned to the P1-I genotype through MLST analysis. The classification inconsistencies observed in this study could be attributed to the ambiguous nature of specific housekeeping gene loci (*ppa*, *gyrB*, *glyA*, and *adk*) within the genome, which consequently lead to indeterminate sequence typing (ST) results. The NJMP202311 genome consistently formed a distinct and independent branch in the phylogenetic tree, irrespective of the analytical approach. Moreover, the genomic characteristics of the CARDS toxin gene, along with the *hmw1*, *hmw2*, and *hmw3* genes, were demonstrated to align closely with those of the P1-I genotype. Further in-depth analysis of its P1 genes indicated that the genome harbors a unique combination of features typically associated with P1-I and P1-II genotypes, suggesting potential evolutionary or functional implications warranting further investigation. However, it should be emphasized that the NJMP202311 genome assembly demonstrated limited completeness (89.41%) and harbored 9,588 ambiguous nucleotides, which could potentially account for the analytical inconsistencies. Due to the lack of a pure cultured strain in this study, more precise sequencing of *M. pneumoniae* in this sample could not be achieved. Therefore, future research should prioritize strain isolation and cultivation, while also investigating whether similar observations occur in other samples.

Several limitations of this study must be acknowledged. Firstly, short-read sequencing data may be inadequate for resolving known repetitive regions within *M. pneumoniae*, potentially resulting in misassembly, over- or under-prediction of coding sequences, and the inability to capture regions utilized in conventional typing schemes. Secondly, the lack of pure *M. pneumoniae* cultures prevented the validation of antibiotic resistance phenotypes and virulence, particularly the differences in CARDS toxin-related genes and adhesion-related genes (*hmw1*, *hmw2*, and *hmw3*) between P1-I and P1-II genotypes. Thirdly, as a cross-sectional investigation, no retrospective analysis was conducted on historical *M. pneumoniae* infection samples in Nanjing. Additionally, the geographical distribution of collected samples was relatively limited due to research constraints. Future studies are required to expand the sample size, broaden sampling regions, and establish long-term surveillance mechanisms to validate further and refine the findings of this research. To validate and refine the current findings, future studies should implement the following improvements: (1) expand both sample size and geographical coverage while establishing systematic long-term surveillance; (2) adopt multicenter study designs to enhance geographical representativeness; (3) optimize sampling protocols by employing diverse isolation and culture techniques to obtain pure bacterial strains; (4) conduct in-depth verification using long-read sequencing technologies coupled with *in vitro* functional assays.

## Data Availability

The datasets presented in this study can be found in online repositories. The names of the repository/repositories and accession number(s) can be found in the article/Supplementary material.

## Footnotes

^1^ https://github.com/s-andrews/FastQC?tab=readme-ov-file

^2^ https://github.com/Ecogenomics/CheckM

^3^ https://github.com/tseemann/barrnap

^4^ https://www.ncbi.nlm.nih.gov/

^5^ https://www.bv-brc.org/

^6^ https://www.cncb.ac.cn/

^7^ https://github.com/tseemann/mlst

^8^ https://pubmlst.org/organisms/mycoplasma-pneumoniae

^9^ https://online.phyloviz.net/index

^10^ https://www.mgc.ac.cn/VFs/

^11^ https://github.com/tseemann/abricate

^12^ https://card.mcmaster.ca/about

^13^ https://github.com/GSLBiotech/mafft

^14^ https://github.com/tseemann/snippy

^15^ https://itol.embl.de/

^16^ https://weblogo.berkeley.edu/logo.cgi

^17^available at: https://github.com/sanger-pathogens/Roary/tree/master/contrib/roary_plots
