# Supplementary figures and images for: Whole-genome probe capture sequencing reveals genomic diversity and characteristics of Mycoplasma pneumoniae in Nanjing, China
Source: Front Microbiol. 2025 May 14;16:1589971. doi: 10.3389/fmicb.2025.1589971 (PMC12116587; doi:10.3389/fmicb.2025.1589971)

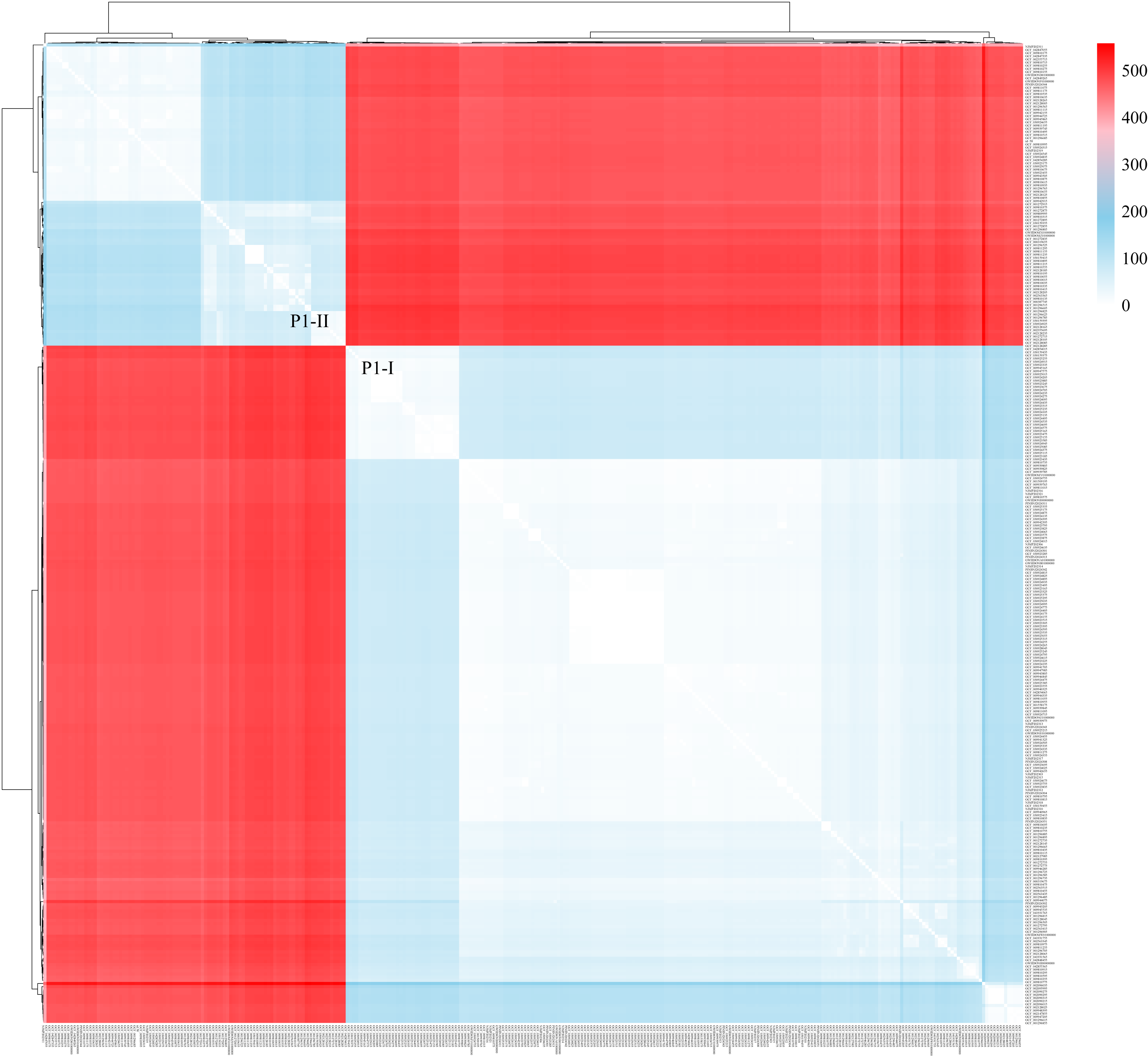

Supplement: Supplementary file 1 [file Image_1.tif]
